# Genome-wide investigation of outer membrane protein families under mosaic evolution in *Escherichia coli*

**DOI:** 10.1128/aem.00557-25

**Published:** 2025-05-30

**Authors:** Xin Cao, Cuihua Cao, Zefan Chen, Jialin Li, Zikun Yao, Yidong Zheng, Jinjin Wu, Zeling Li, Yueming Hu, Gaofeng Hao, Guoqiang Zhu, Wolfgang Köster, Aaron P. White, Yejun Wang

**Affiliations:** 1 Youth Innovation Team of Medical Bioinformatics, Shenzhen University Medical School 477382, Shenzhen, Guangdong, China; 2 Department of Bioinformatics, College of Life Sciences, Zhejiang University 98445 , Hangzhou, Zhejiang, China; 3 Department of Stomatology, Shenzhen Maternal and Child Health Care Hospital Affiliated to Southern Medical University, Shenzhen, China; 4 College of Veterinary Medicine, Yangzhou University 614704, Yangzhou, Jiangsu, China; 5 Vaccine and Infectious Disease Organization-International Vaccine Centre 483123 , Saskatoon, Saskatchewan, Canada; 6 Department of Cell Biology and Genetics, College of Basic Medicine, Shenzhen University Medical School 477382, Shenzhen, Guangdong, China; Centers for Disease Control and Prevention, Atlanta, Georgia, USA

**Keywords:** mosaic evolution, outer membrane protein (OMP), highly variable region (HVR), positive selection, *Escherichia coli*

## Abstract

Several genes in Gram-negative bacteria encoding outer membrane proteins (OMPs) have been reported to show patterns of mosaic evolution featured by a mixture of negative selection and local recombination. Here, we improved a strategy and applied it to screen OMPs under mosaic evolution in *Escherichia coli* at the genome level. In total, 21 OMP families, including 16 new ones, were detected with the typical patterns of mosaic evolution. An absolute majority of the protein families are conserved in *E. coli* for the composition, genomic loci, and the overall structures. Highly variable regions (HVRs) can be recognized, which are frequently located extracellularly within the protruding loops. There is only a limited number of major HVR sequence types, within which positively selected sites can be detected occassionally. Based on the simulated results of multiple models, the OMPs under mosaic evolution are often with good antigenicity, with HVRs of various sequence types coinciding with the B-cell epitopes of the strongest immunogenicity. The study further broadened our understanding of the characteristics of mosaic evolution and the functions of OMPs in Gram-negative bacteria, laying an important foundation for their potential translational applications.

**IMPORTANCE:**

It is important to understand the evolutionary mechanisms of bacterial OMP-encoding genes, which would facilitate the development of anti-bacterial reagents. This study made the first genome-wide screening of bacterial OMPs under mosaic evolution and increased the list of candidate OMP families by threefold in *E. coli*, far more than we expected. The study further confirmed the hypothesis about the evolutionary, micro-evolutionary, and structural features of these OMPs and facilitated the functional theory of mosaic evolution. Moreover, the findings of limited HVR sequence types and strong immunogenicity of HVRs paved an important foundation for the application of these OMPs and their HVRs in the development of antibodies or other antibacterial treatment.

## INTRODUCTION

Bacterial infection is a severe threat to human health, while antimicrobial resistance (AMR) worsens the situation, leading to millions of deaths globally every year (1). It is urgent to develop new strategies to cope with bacterial infections. On the one hand, there is a need to accelerate the development of novel antibiotics (2–5), therapeutic monoclonal antibodies (6), and other treatment methods (7), such as bacteriophage therapy (8). On the other hand, it is also crucial to deepen our understanding of the mechanisms of bacterial infection, pathogenicity, and AMR from the perspectives of bacterial evolution and selection (9, 10) and to improve the susceptibility of bacteria to various existing antibacterials (11).

The outer membrane proteins (OMPs) are closely related with bacterial pathogenicity and AMR and also important targets for the development of antimicrobial reagents (7, 12). Gram-negative bacteria have two layers of cell membrane, outer membrane and inner membrane, enclosing the periplasm space (13). The OMPs span the outer membrane in Gram-negative bacteria, playing important roles in signaling and transmembrane transport of molecules (7, 12). Due to exposure at the cellular surface, the extracellular portions of bacterial OMPs often serve as target molecules for antibacterial factors such as immune factors, bacteriophages, and antibiotics. Therefore, in recent years, some novel therapeutic monoclonal antibodies (14–16) and phage therapies (17, 18) were developed, which often target bacterial OMPs.

Bacteria themselves have also evolved various strategies, such as altering the sequences and structures of the OMPs or downregulating their expression, to evade the attacks of these naturally occurring or artificially designed antibacterial factors, resulting in phenotypes such as immune evasion and bacteriophage tolerance (19, 20). Exploring the evolutionary characteristics and patterns of bacterial OMPs can aid in designing more effective antibacterial agents. Evolutionary studies indicate that bacterial OMP genes, such as *fhuA*, *lamB*, *ompA*, *ompC*, *ompF*, and others, are often under positive selection (21, 22), possibly due to their frequent targeting by bacteriophages, immune factors, and other disfavoring factors in the environment (23, 24). In the laboratory, dynamic sequence variation in *lamB*, *ompA*, and *ompF* can be directly detected by exposing *E. coli* to phage infestation, confirming large evolutionary rates of these OMP genes (9). These evolutionary characteristics of OMP genes allow bacteria to rapidly derive and select clones tolerant to adverse factors in the current environment, promoting population survival. These evolutionary traits of bacterial OMPs also accelerate the failure of antibiotics, vaccines, and other treatments, thereby increasing the difficulty of antibacterial therapy (9, 25).

Previously, we found that the *fhuA* gene exhibits significant intra- or inter-species local recombination in *E. coli* and *Salmonella* (23). The local recombination regions, also known as highly variable Rrgions (HVRs), are generally concentrated. FhuA serves as a binding receptor for multiple bacteriophages, and its HVRs are highly consistent with the binding sites of bacteriophages, suggesting that *fhuA* avoids bacteriophage invasion through local recombination (23). Within the population of *E. coli* with the same HVR type, there is also a certain degree of positive selection within the HVRs (21–23). Apart from the HVRs, other sequences of FhuA are highly conserved, displaying purifying selection; correspondingly, the overall structure of FhuA proteins across different HVR sequence types is similar, which may be related to the maintenance of the basic function of FhuA, i.e., transporting iron chelates. Subsequently, we further disclosed multiple porin genes in *E. coli*, including *lamB*, *ompA*, *ompC,* and *ompF*, which exhibit the typical patterns of mosaic evolution similar to *fhuA*, characterized by the coexistence of local recombination, positive selection, and negative selection (24). There are OMP genes in other bacteria which were reported to show similar evolutionary patterns (26–29). Based on these observations, we proposed the mosaic evolution theory of bacterial OMPs. Mosaic evolution is defined by an OMP with most sequence fragments conserved, interspersed with local regions frequently undergoing ingenic recombination and positive selection. By this evolutionary means, bacteria maintain the overall protein structure and function through the conservation of genes and overall sequences, while promoting resistance to bacteriophage and other adverse factors through local recombination and positive selection (23, 24).

To further analyze the distribution of mosaic evolution in bacterial OMPs, in this study, we improved the analytical methods to make genome-wide screening of the OMPs under mosaic evolution in *E. coli*. For the candidate proteins, we further delineated and characterized the HVRs, observed the HVR sequence types, distribution and micro-evolution, and analyzed their structures and immunogenicity.

## RESULTS

### Mosaic evolution of OMP-encoding genes in *E. coli*

**Fig 1:**
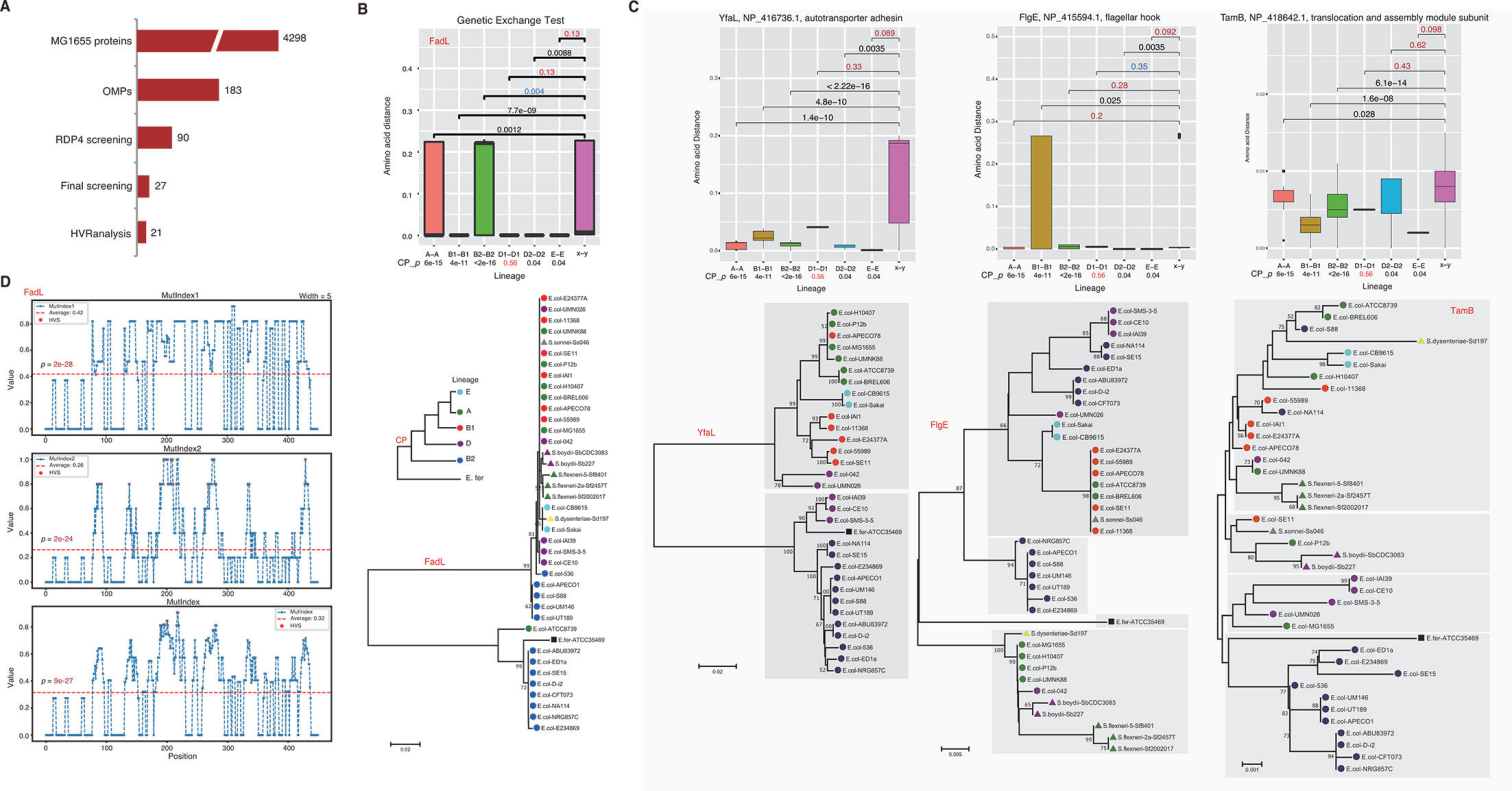
Screening of *E. coli* OMPs under mosaic evolution. (**A**) The OMPs and candidates under mosaic evolution detected from each step. (**B**) Genetic exchange test and phylogenetic clustering for FadL protein families. The genetic distance among *E. coli* strains within each lineage was shown and compared to that among strains from different lineages, respectively. Mann-Whitney *U* tests were performed, and *P*-values larger than 0.05 are highlighted in red. The significant *P*-values but with larger intra-lineage distance are shown in blue. A-A, B1-B1, B2-B2, D1-D1, D2-D2, or E-E represent strain pairs from the same lineage: A, B1, B2, D1, D2, or E, respectively. “x-y” represents pairs of strains from different lineages, e.g., A-B1, A-B2, D1-D2, etc. The *P*-values for core-proteome (CP_*p*) were shown at the bottom. FadL proteins were clustered by the neighbor-joining tree. The robustness of the phylogenetic tree was examined by bootstrapping tests with 1,000 replicates, and the scores are indicated for nodes of no lower than 50%. The strains from the same *E. coli*, *Shigella,* or *E. fergusonii* lineage are labeled with a unique colored sign. The diagram for core-proteome (CP) tree referred to (24) and was also shown. (**C**) Genetic exchange tests and phylogenetic clustering for other representative OMP families. The different phylogenetic clusters were shown in rectangles with a gray background. (**D**) Distribution of three indices reflecting the local variation property of the FadL protein family in *E. coli. HVR analysis* was used for the analysis with the window width and step size set as 5 and 1, respectively. The significant highly variable sites (HVS) were shown in red circles.

We predicted 183 OMPs from the 4,298 genome-derived proteins of *E. coli* strain MG1655 and identified their orthologs from other *E. coli* strains and *E. fergusonii* ATCC_35469 (Fig. 1A). With RDP4 (version 4.101), we found that 90 OMP-encoding gene families experienced ingenic local recombination (Fig. 1A; Data set S1). Phylogenetic trees were built and manually examined for these genes and the proteins, to further screen those incongruent with the lineage tree for both types of sequences. Genetic exchange tests were also performed to confirm the local ingenic recombination for each of the gene and protein families. The procedure detected 27 genes showing typical mosaic evolution patterns at both nucleotide and amino acid levels (Fig. 1A; Data set S1).

We took *fadL* as an example to demonstrate the mosaic evolution (Fig. 1B and C). FadL is a long-chain fatty acid transporter, which is also a receptor for bacteriophage T2 (Data set S2). For some lineages, for example, lineages D1 and E, the protein sequences of the *fadL* gene showed no significant difference between the within-lineage distance and the inter-lineage distance (Fig. 1B). In some cases, such as the lineage B2, the FadL protein sequences even showed significantly larger distance among strains within the same lineage than the inter-lineage distance (Fig. 1B). As a control, however, the distance of *E. coli* core proteome was significantly larger between lineages than within the same lineages expect for D1, which showed no significant difference (Fig. 1B). The FadL protein tree showed apparent incongruence with the *E. coli* lineage tree based on the core proteome, featured by the divergence and cluster separation of proteins from the strains of the same lineages (e.g., MG1655 and ATCC8739 of lineage A and S88 and ED1a of lineage B2), or the convergence of proteins from the strains of different lineages (e.g., ATCC8739 of lineage A and ED1a of lineage B2) (Fig. 1B). The genetic exchange analysis and phylogenetic analysis on *fadL* nucleotide sequences showed consistency with the results on the protein sequences. Taken together, the results demonstrated that the *E. coli fadL* gene experienced events of ingenic recombinations. The results of genetic exchange tests and phylogenetic analysis were also shown for other three representative proteins, FlgE (a flagellar hook protein), YfaL (an autotransorter adhesin), and TamB (a translocation and assembly module subunit protein), all suggesting typical mosaic evolution patterns (Fig. 1C).

We further proposed three indices, namely, MutIndex1, MutIndex2, and MutIndex, to illustrate intuitively the property of local mutations of the proteins under mosaic evolution, to test the unevenness of mutation along the protein sequence, and to identify HVRs and the sequence types of each HVR (Materials and Methods). With this strategy, among the 27 *E. coli* OMP families, 21 demonstrated striking mosaic evolution patterns (Fig. 1A).

For each protein, the three indices showed similar distribution patterns along the protein sequence, all of which were significantly different from random distributions, confirming the mosaic evolution (Fig. 1D, FadL as an example). According to the distribution patterns of the indices, especially the composite index MutIndex, local fragments (HVRs) could be delineated, for which the MutIndex values were strikingly larger than the average values (Fig. 1D). The HVRs and their sequence types and subtypes were annotated for all the 21 protein families, where an HVR sequence type was defined as more than two point mutations (including insertions/deletions), while a subtype was defined as no more than two point mutations for the HVR sequences between each other (Data set S1).

### Overall conservation of *E. coli* OMP families under mosaic evolution

**Fig 2:**
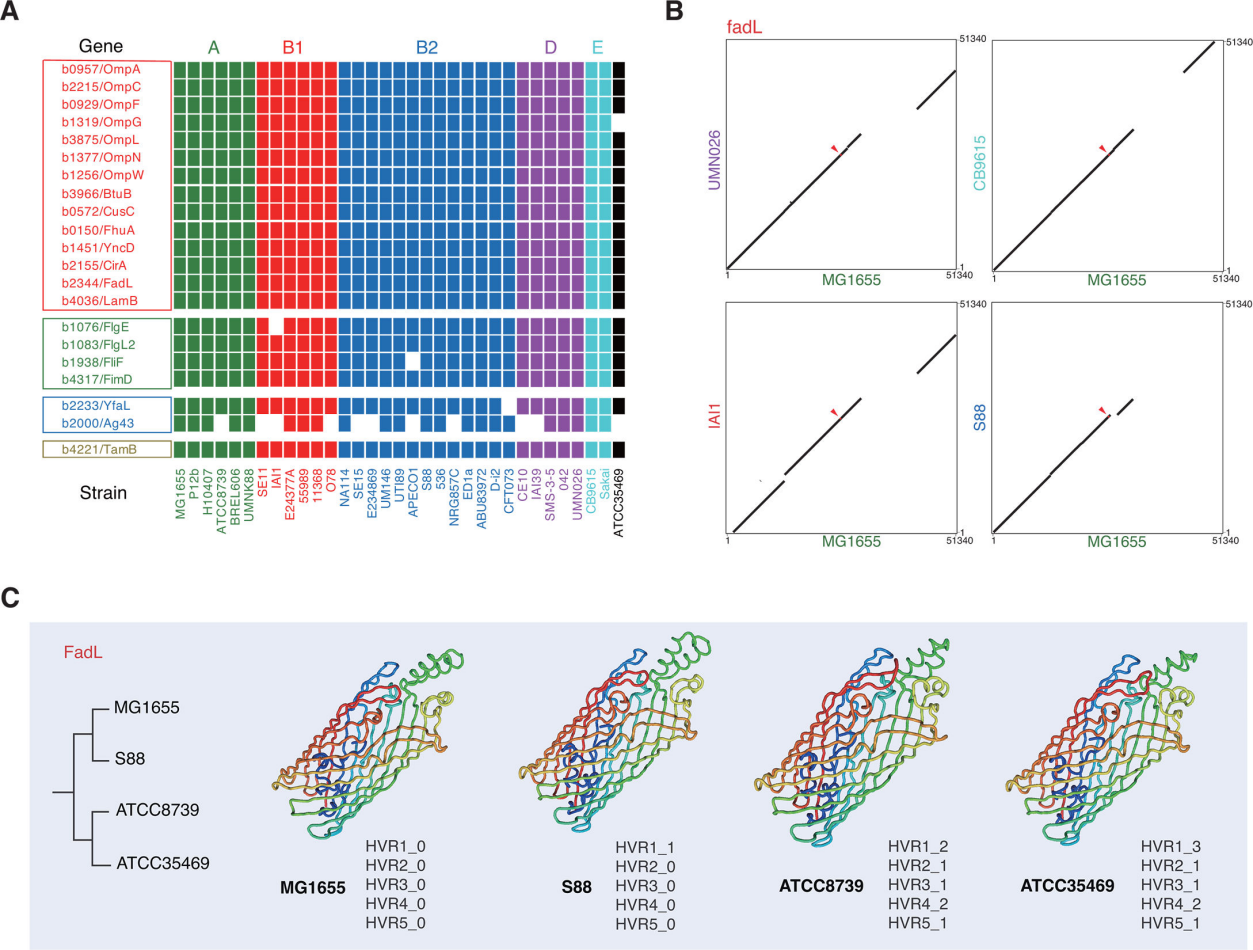
Conservation of *E. coli* OMPs under mosaic evolution. (**A**) Distribution of OMPs under mosaic evolution in *E. coli* strains. Missing a gene in a strain was shown in white, while the presence was shown in different colors for different lineages. Genes were grouped according to their main functions, including beta-barrel proteins, flagella and fimbriae components, adhesins and membrane-attaching antigens, and other transmembrane functional factors. (**B**) Collinearity of the *fadL* gene and the flanking nucleotide sequences (25 kb at each side) between *E. coli* strains from different lineages. (**C**) The tertiary structures of FadL proteins of representative *E. coli* strains from different sequence clusters. The N-terminal 1-25 residues for each FadL protein that were predicted to be signal peptide by SignalP 6.0 were removed. The sequence types of the FadL HVRs were indicated under each structure. The detailed sequences for the HVRs were annotated in Supplemental Data set S1.

The 21 candidate *E. coli* OMPs under mosaic evolution were functionally annotated in Data sets S2. Most (14/21) of them encode porins or other transmembrane channels (Fig. 2A), including the known families of FhuA, LamB, OmpA, OmpC, and OmpF (23, 24). Some flagella or pilus components (4/21), Type V autotransported adhesins or antigens (2/21), and factors functioning on the membrane (1/21) also show the patterns of mosaic evolution (Fig. 2A). A majority of the genes have essential functions for bacterial cells and are conserved among *E. coli* lineages and strains, except for *ag43*, which showed poor conservation (Fig. 2A). Ag43 is a two-part type V autotransported surface antigen, but the exact function remains unclear. For each of the other OMP families detected with mosaic evolution, each *E. coli* strain contains no more than one gene copy, and the genetic loci show good collinearity among different *E. coli* lineages (Fig. 2B). The *ag43* loci also show apparent collinearity among the strains bearing the gene. Therefore, the OMP genes under mosaic evolution are generally conserved.

Tertiary structure of the proteins was also modeled and compared. Generally, the structure of full-length proteins appeared conserved. FadL was shown as the representative example. The structure of the mature FadL proteins (with signal sequences removed) from different phylogenetic clusters always appeared quite conserved between each other, all close to the experimentally resolved structure of FadL (30). The proteins from different phylogenetic clusters have different HVR sequence types or varied combinations, implying that the basic function of the proteins was not likely disrupted by the local HVR recombinations (Fig. 2C).

### Transmembrane topology and structure of the *E. coli* OMP HVRs

**Fig 3:**
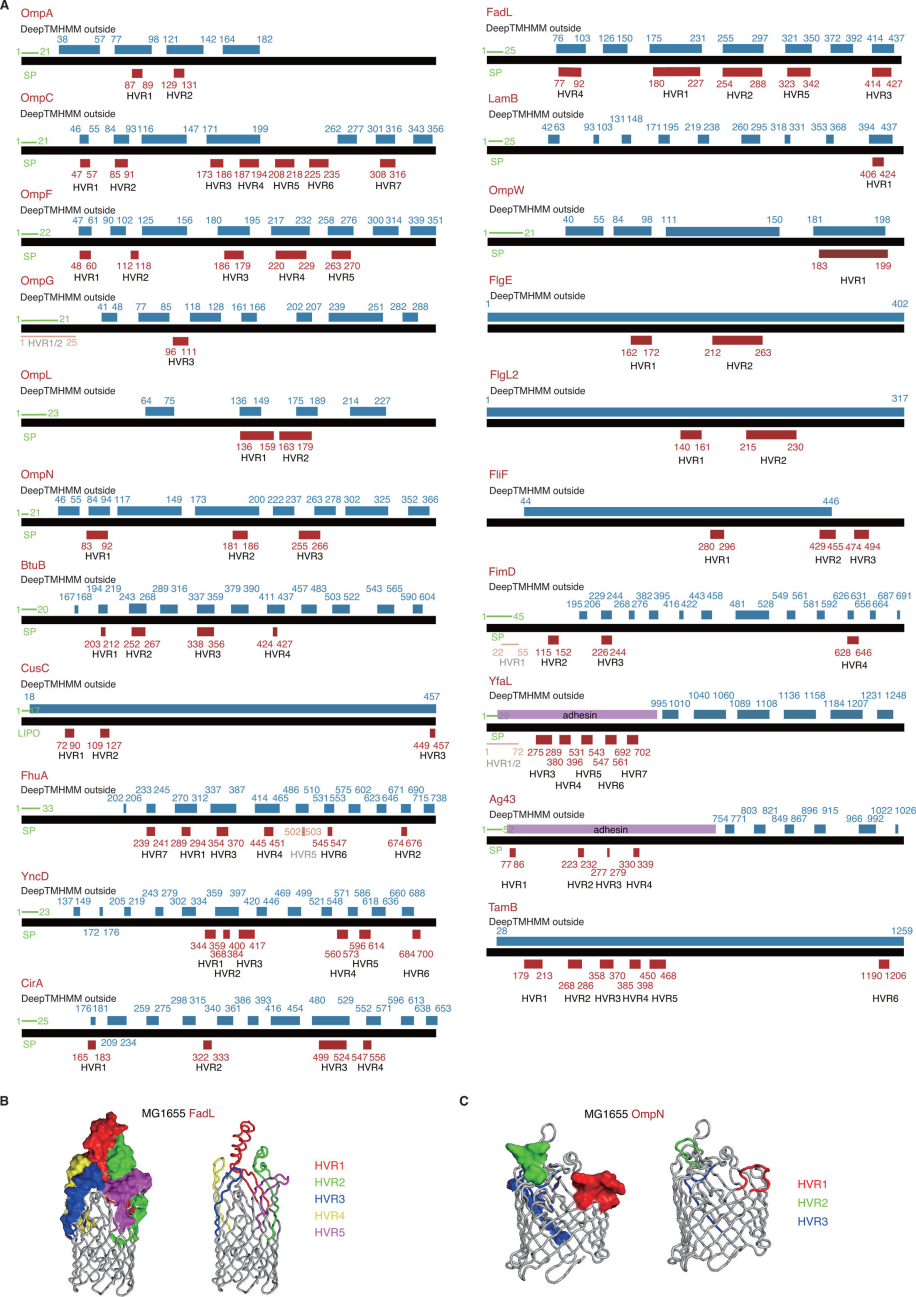
Transmembrane topology and tertiary structure of the *E. coli* OMPs under mosaic evolution. (**A**) Transmembrane topology diagrams and the location of HVRs for the 21 *E. coli* OMPs under mosaic evolution. The signal peptides were predicted with SignalP 6.0 and shown in green. The transmembrane topology was predicted with DeepTMHMM for the mature proteins with signal peptides removed, and the extracellular segments were shown in blue. The HVRs identified by *HVR analysis* and shown in red. The DeepTMHMM prediction results were corrected with the tertiary structure that was experimentally resolved or predicted. The adhesins were annotated manually from literature but not predicted to be extracellular segments by DeepTMHMM, and they were also corrected and shown in purple. The positions indicated for the start and end for each segment were based on the full-length proteins with signal peptides. (**B**–C) The tertiary structure of FadL (B) and OmpN (C), and the location of HVRs. The signal peptides were removed before the structure modeling and illustration. The *E. coli* MG1655 proteins were used for all the analysis and structure illustration.

The transmembrane topology was predicted with DeepTMHMM on the OMPs under mosaic evolution. As expected, the HVRs often coincide with the extracellular fragments for these proteins (Fig. 3A). OmpG and YncD are exceptions for which the HVRs do not overlap with the extracellular fragments (Fig. 3A). Some HVRs are located in the signal peptides predicted by SignalP, such as the N-terminal HVRs in OmpG, FimD, and YfaL (Fig. 3A). It would be interesting to know whether the variations could influence the secretion of proteins through the inner membrane. It is also unclear about the significance of local recombinations within the signal peptide regions. It should also be pointed out that the N-terminal halves of YfaL and Ag43 were not predicted as extracellular fragments by DeepTMHMM, while the HVRs are all located in the N-terminal halves of these proteins (Fig. 3A). Database and literature annotation both suggested that both YfaL and Ag43 are autotransported adhesins, while the N-terminal halves are exported extracellularly through the outer-membrane conduit formed by the C-terminal halves of the proteins (Fig. 3A; Data set S2).

Consistent with the topology, the tertiary structure modeled for these proteins also suggests the frequent extracellular exposure of the HVRs. For example, all the HVRs of FadL are located in the surface of extracellular loops (Fig. 3B). Similarly, the HVRs of OmpN are also located in the protruding regions of the extracellular protein loops (Fig. 3B). Many of these proteins, including FadL, OmpW, and even flagella-related proteins, have been reported to serve as binding receptors of various bacteriophages (Data set S2). Previous observations on FhuA, LamB, OmpA, OmpC, and OmpF suggest that the HVRs could also potentially be the phage-binding sites for these proteins (23, 24).

### Limited sequence types of the OMP HVRs in *E. coli* strains

**Fig 4:**
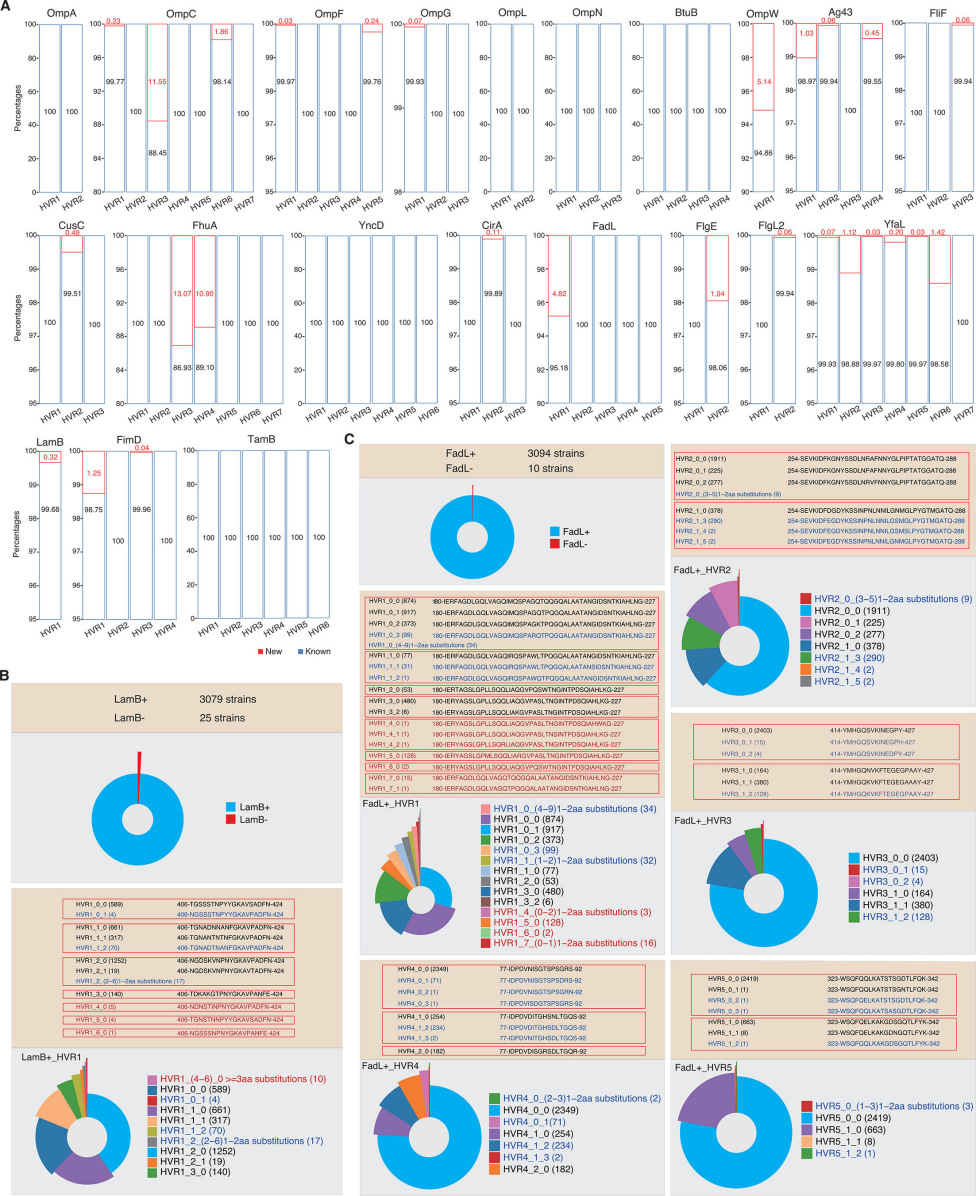
Distribution of HVR sequence types for each OMP under mosaic evolution in *E. coli* strains. (**A**) Distribution of HVR sequence types for the 21 *E. coli* OMPs under mosaic evolution in 3,104 independent *E. coli* strains. Proportions of newly defined HVR types were shown in red, while the known ones (from the 40 training strains) were shown in blue. (**B**) Distribution of LamB HVR sequence types and subtypes in the independent *E. coli* genome set. (**C**) Distribution of FadL HVR sequence types and subtypes in the independent *E. coli* genome set. The sequences for the new HVR types and HVR subtypes were shown in red and blue, respectively. The positions were based on the real coordinates of the HVRs within the full-length proteins with signal peptides of *E. coli* MG1655.

According to the definition, OMP-encoding genes under mosaic evolution should experience local recombinations more often for the HVRs, and the sequence types for most HVRs are hypothesized to be with a limited number. We, therefore, resolved the HVRs and observed the distribution of HVR sequence types in the corresponding proteins among an enlarged size of *E. coli* strains (Data set S3). Except for only a few HVRs, for example, HVR3 of OmpC, HVR3 and HVR4 of FhuA, HVR1 of OmpW, and HVR1 of FadL, no or very few strains showed new HVR sequence types (Fig. 4A; Data set S3). Even though there are strains showing new HVR sequence types, the number of new types is very limited (Data set S3).

We further took LamB and FadL as examples, which have one and multiple HVRs, respectively, to demonstrate the distribution of new HVR sequence types and subtypes. LamB was detected from 3,079 (99%) out of the total 3,104 representative *E. coli* genomes (Fig. 4A). One HVR (HVR1) and four major sequence types (HVR1_0 ~ 3) were detected from the 40 *E. coli* or close strains used for mosaic evolution screening in this study, while each major HVR sequence type contains 1–2 subtypes with 1–2 amino acid substitutions (Fig. 4B). Among the 3,079 LamB-positive strains, there are three new major HVR sequence types detected (Fig. 4B). However, their new HVR sequence types only cover 10 strains (0.3%) in total (Fig. 4B). 96.7% (2978/3079) of the strains showed the HVR sequence subtypes exactly consistent with the known ones, while only ~3.0% (91/3079) showed only 1–2 amino acid substitutions compared to the known sequence subtypes, respectively (Fig. 4B).

Nearly 99.7% (3,094/3,104) of the *E. coli* genomes could be detected with the FadL-encoding gene (Fig. 4C). For the 5 HVRs of FadL proteins, HVR1 ~5 each contains 4, 2, 2, 3, 2 major sequence types, respectively (Fig. 4C). Except for HVR1, no novel major sequence type was detected from the 3,094 FadL-positive *E. coli* strains for each HVR (Fig. 4D through H). Four new major sequence types were detected for HVR1 from 149 strains, among which a major type with a unique sequence is responsible for 128 strains, while another one is responsible for 15 strains (Fig. 4C). For HVR1 ~5, there are 89.9% (2,780/3,094), 90.2% (2,791/3,094), 95.2% (2,947/3,094), 90.0% (2,785/3,094), and 99.9% (3,090/3,094) of the strains showing sequence subtypes exactly the same as the known ones (Fig. 4C).

Taken together, the results demonstrated that the number of major sequence types for the HVRs of OMPs under mosaic evolution is quite limited. Natural mutations also happen but slowly within the HVRs.

### Micro-evolution of the HVRs of *E. coli* OMPs under mosaic evolution

**Fig 5:**
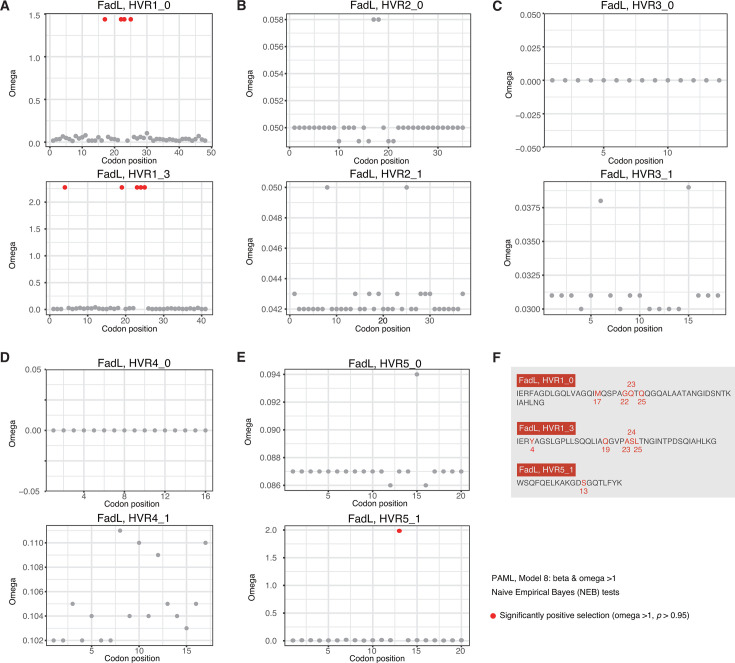
Selection pressure of FadL HVRs in *E. coli*. (**A–E**) Omega distribution along the sites of each major HVR sequence type of FadL in *E. coli*. The sites of significant positive selection were shown in red. (**F**) Summary of the positively selected sites detected in *E. coli* FadL HVRs. PAML was used for the selection analysis.

We calculated the selection pressure for each major sequence type of the HVRs of FadL in *E. coli*. Despite the non-positive selection for the whole fragments of the HVRs and most codon positions, several sites were detected with significantly positive selection (Fig. 5A through E). Specifically, the 19th, 22nd, 23rd, and 25th codons of HVR1_0, the 4th, 19th, 23rd, 24th, and 25th codons of HVR1_3, and the 13th codon of HVR5_1 were all positively selected (Fig. 5A, E and F). Previously, we also detected a few sites under significant positive selection within LamB and OmpC HVRs (24). Therefore, the HVRs for individual sequence types could also experience positive selection, albeit slowly, so as to make sequence varying, generating new major HVR sequence types eventually.

### Immunogenicity of the HVRs of *E. coli* OMPs under mosaic evolution

The HVRs could be good antigens due to their extracellular localization, hydrophilicity, and relative conservation of the sequence types. Consequently, we tested their potential immunogenicity. LamB and OmpA, two proteins documented to be possible good candidates for vaccine or antibody development (15, 19), were selected as representatives for analysis.

**Fig 6:**
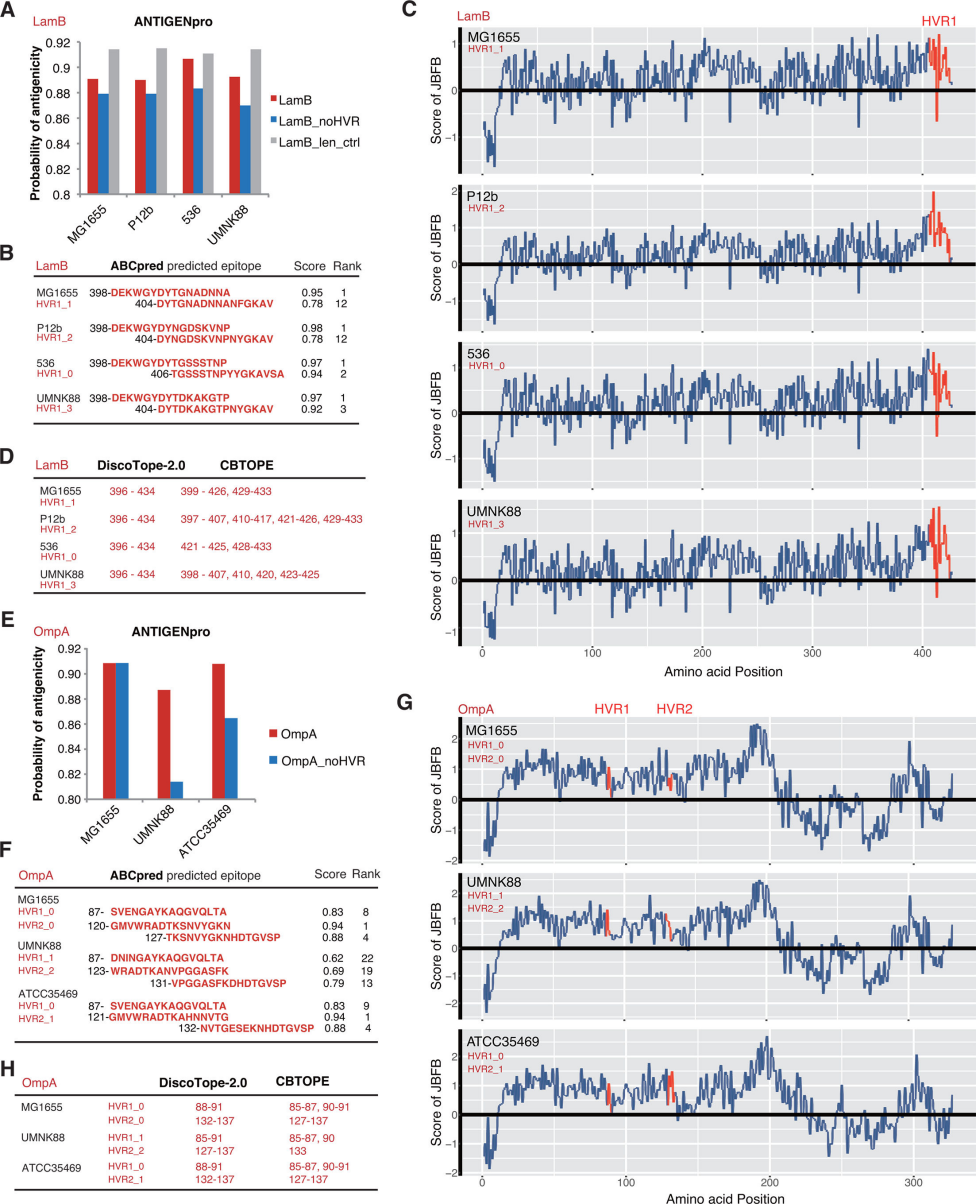
Immunogenicity of *E. coli* LamB and OmpA HVRs. (**A**) Antigenicity of LamB of different sequence clusters. LamB, LamB_noHVR, and LamB_len_ctrl represented the wild-type mature LamB with the signal peptides revmoved (residues 1–25 of the original full-length proteins), HVR-removed mature LamB, and mature LamB removed of non-HVR fragments with the total length identical to that of the HVRs (residues 26–44 of the original full-length proteins), respectively. (**B**) The scores and ranks of ABCpred predicted B-cell epitopes in LamB HVRs. (**C**) The predicted scores of JBFB on the linear B-cell epitopes in LamB proteins from different sequence clusters. (**D**) Structural B-cell epitopes in LamB HVRs predicted by DiscoTope-2.0 and CBTOPE. (**E**) Antigenicity of OmpA of different sequence clusters. OmpA and OmpA_noHVR represented the wild-type mature OmpA (with the signal peptides removed) and HVR-removed mature OmpA, respectively. (**F**) The scores and ranks of ABCpred predicted B-cell epitopes in OmpA HVRs. (**G**) The predicted scores of JBFB on the linear B-cell epitopes in OmpA proteins from different sequence clusters. (**H**) Structural B-cell epitopes in OmpA HVRs predicted by DiscoTope-2.0 and CBTOPE. For (**B**), (**C**), (**D**), (**F**), and (**H**), the full-length proteins containing signal peptides were used for analysis and numbering the HVR locations. For JBFB, the window size for the epitopes was set as 20 by default.

LamB of each HVR sequence type was predicted to have high antigenicity with an ANTIGENpro probability of >0.88 (Fig. 6A). The antigenicity of control LamB proteins with HVRs removed (LamB_noHVR) was reduced apparently compared to the wild-type LamB proteins (LamB) or LamB proteins removed of the same length of non-HVR fragments (LamB_len_ctrl), suggesting the important contribution of HVRs to the antigenicity of LamB proteins (Fig. 6A). It should be noted that all the LamB proteins without the HVR still showed a high probability of antigenicity (>0.85), also indicating the important contribution of the fragments outside the HVR (Fig. 6A). Linear B-cell epitopes were further predicted from LamB proteins with an online tool ABCpred and JBFB, a newly developed tool with different features and algorithms (Materials and Methods). With ABCpred, we detected two significant B-cell epitopes from each protein, which overlap with the HVR of LamB (Fig. 6B). For all four LamB proteins with different HVR sequence types, the most significant B-cell epitopes are with the same location in the HVRs (Fig. 6B). The results also suggested the likely immunogenic contribution of the fragments nearby, especially to the N-termini of the HVRs (Fig. 6B). However, a previous mutagenesis study emphasized more the immunogenic contribution of sites (407, 411, 412, 419) within the HVR of LamB (19). Consistent with ABCpred and the previous experimental study, JBFB predicted that the HVR of MG1655 showed the strongest immunogenicity along the LamB protein though the fragments to the N-terminus of the HVR also showed strong immunogenicity (Fig. 6C, red). The LamB proteins of different HVR sequence types showed similar results (Fig. 6C). Furthermore, DiscoTope-2.0 and CBTOPE were used for the prediction of structural B-cell epitopes. Consistent with the observation for linear epitopes, both tools predicted significant structural B-cell epitopes from LamB proteins of different sequence types, all being located in and nearby the HVRs (Fig. 6D).

Similar observations were obtained from OmpA. The OmpA proteins of different HVR sequence types all showed a large probability of antigenicity, with UMNK88 OmpA showing a relatively lower antigenicity (Fig. 6E). The antigenicity decreased strikingly for two OmpA proteins with HVRs removed (UMNK88 and ATCC35469 OmpAs), implying the significance of HVRs (Fig. 6E). No difference in antigenicity was observed from the MG1655 OmpAs with and without HVRs, while the UMNK88 and ATCC35469 OmpAs without HVRs also showed high probability of antigenicity, suggesting the important contribution of other peptide fragments (Fig. 6E). Consistent with ANTIGENpro results, the prediction results from ABCpred showed significant B-cell epitopes within the two HVRs of OmpA proteins with different HVR sequence types and a little weaker immunogenicity of UMNK88 OmpA (HVR1_1 and HVR2_2) (Fig. 6F). JBFB also recognized the positive epitopes from both HVR1 and HVR2 in all three OmpA proteins though the prediction scores were not the largest along the proteins (Fig. 6G). Both DiscoTope-2.0 and CBTOPE also predicted significant structural B-cell epitopes within the two HVRs of OmpA proteins of different HVR types (Fig. 6H).

Taken together, the results suggested that the OMPs under mosaic evolution could potentially be used for antibody development. The HVRs of various sequence types could serve as target antigens, which often bear strong immunogenic epitopes.

## DISCUSSION

Several OMP families have been reported to show patterns of mosaic evolution in different bacteria (23, 24, 26–29). However, it is unknown how many OMP families are experiencing such types of evolution in a bacterial species. To our knowledge, this study made the first genome-wide investigation on the mosaic evolution in OMP-encoding genes in *E. coli*. To our surprise, around half of the OMP genes in *E. coli* can be detected with ingenic recombination events, as predicted by RDP4 (Data set S1). Many of the recombinations generate no or only atypical changes in protein level. However, there remain 21 OMP families showing typical patterns of mosaic evolution, including the incongruence of gene or protein trees featured by inter-lineage convergence and clustering, larger within-lineage but smaller inter-lineage molecular distance, and being detected with significant uneven fragmental mutation rates and typical HVRs (Data set S1). The five known porins under mosaic evolution (FhuA, LamB, OmpA, OmpC, OmpF) are all included in the 21 candidate proteins. Therefore, the real number of bacterial OMPs under mosaic evolution could be much larger than we expected. It would be interesting to investigate whether it is the case in other bacteria. While a large majority of the OMPs under mosaic evolution are beta-barrel proteins, we also found that some outer membrane attachments showed similar patterns, including flagellar, fimbrial, and adhesin proteins (Fig. 2). Particularly, in this study, we reported for the first time two Type V secreted proteins (Ag43 and YfaL) of mosaic evolution patterns (Fig. 3A).

Most of the OMPs under mosaic evolution have functions essential for bacterial survival. The proteins from different sequence clusters always show conserved tertiary structures in general (Fig. 2C). With very few exceptions, the genes are present in and genomically conserved among *E. coli* strains (Fig. 2A and B). The proteins are often receptors of phages, immunogenic, and factors responsible for AMR (Data set S2). Previously, we noticed that the HVRs often coincided with the phage-binding sites (23, 24). In this research, we found that the HVRs are also candidate fragments that could elicit strong B-cell immune responses (Fig. 6). Therefore, the theory on bacterial mosaic evolution was further consolidated by this study. Bacteria could rapidly escape from the attacks of harmful factors, such as phages, immune factors, and others, and maintain the essential function by rapid ingenic recombination for the HVRs (24). These HVRs have actually been experiencing a long period of evolution and selection and diverged from their ancestral sequence type very slowly, and therefore, the fragmental conversion does not lead to bacterial fitness problems. In fact, within the major HVR sequence type, we did find, though very few, HVR sites under positive selection, in FadL (Fig. 5), LamB, and OmpC (24). There remains a question for the theory as to the sources of HVRs. It was reported that various recombinations and gene conversions are widely spread in *E. coli* and other bacteria (31, 32) although the recombination sources are also unknown. The microbiota co-present in the environment where the target strain was isolated could serve as an optional library for the genetic molecule sources.

The study also paved the foundation for potential applications of mosaic evolution and HVRs for the development of antibacterial reagents, for example, monoclonal therapeutic antibodies. Some of the proteins screened in this study have been tested in *E. coli* or other species for vaccine development or eliciting antibodies (Data set S2). For example, the HVR-coinciding amino acid sites in *E. coli* LamB appeared crucial for eliciting effective immune responses (19). FadL and especially the extracellular loops were also used for vaccine development, which could elicit effective antibodies against *Treponema pallidum* (33, 34). Traditionally, the conserved peptide fragments rather than the HVRs should be considered stable antigen candidates for antibody development. However, the long-term co-evolution between bacteria and the hosts and the selection have often made the conserved peptide fragments not located extracellularly, unexposed, and lowly immunogenic. The HVRs are located extracellularly, accessible by the immune factors, and with strong immunogenicity. The essential function of the proteins and the conserved presence among strains reduce the risk of off-target. The recombination nature of HVRs ensures the limited number of major sequence types and the predictability (Fig. 4), making it possible to develop a mixed pool of antibodies against each major HVR sequence type. Each HVR sequence type or subtype seemed to show generally strong immunogenicity (Fig. 6). These results all suggest the promising application of these OMPs and HVRs (35). Furthermore, some of the OMPs under mosaic evolution, such as LamB, form trimers in the outer membrane (36), which could increase a complement-mediated bactericidal activity after binding of antibodies to the HVRs. However, the HVRs predicted to be with strong immunogenicity could be ineffective practically. Computational predictions are not always accurate, and the long sugar chains of lipopolysaccharides might mask the mostly smaller extracellular loops of OMPs located close to the outer rim of the membrane (14). Therefore, the practical effect should be observed with more elaborate and extensive experimental validation. It would also be important to test whether an antibody against an HVR subtype could cross-react with other subtypes of the same major sequence type with 1–2 amino acid variations.

Annotation of the *E. coli* OMPs under mosaic evolution suggested that more than half of them, including OmpA, LamB, OmpC, OmpF, OmpW, OmpN, FhuA, CirA, CusC, FlgE, and FimD, were reported to be associated with AMR (Data set S2). It would be interesting to know whether the mutations or recombinations of the genes encoding these OMPs could lead to AMR directly. Our preliminary analysis suggested that the HVR sequence types for some of these OMPs correlate with the AMR phenotype in *E. coli*. Further mechanistic investigations are desired, which may disclose novel insights on the molecular mechanisms of bacterial AMR.

Finally, we should also point out the possible limitations of the current study. The OMPs were only predicted from *E. coli* MG1655 but could not represent the full set in *Escherichia*. There could be more OMPs in *Escherichia* but missed from MG1655. The OMPs predicted by computational tools could not be absolutely accurate. Some real OMPs could be missed, and also some predicted OMPs are actually non-OMPs. Even though a manual check was performed on the predicted OMPs, some false positives, though very few, could still be inevitable. For example, TamB was predicted to be an OMP by different computational tools, but databases and literatures showed divergence, with some supporting it to be an OMP and others considering it to be a non-OMP (Data set S2). Similarly, DeepTMHMM is widely used for transmembrane topology analysis and generally accurate. For example, the transmembrane topology predicted by DeepTMHMM of the mature FadL protein showed good consistency with the reported crystal structure (30). However, the tool also showed limitations. It could not accurately predict the extracellular fragments of the adhesins, Ag43 and YfaL (Fig. 3A). In this study, we also compared the DeepTMHMM prediction results with the experimentally resolved or RoseTTAFold predicted tertiary structures and referred to the publications or databases annotating the proteins, so as to correct the prediction results. However, the structure and function for some proteins remain unclear, and the transmembrane topology could not be consolidated. The tools predicting antigenicity and immunogenicity could have similar problems. Taken together, the the study could provide some interesting observations and insights on the evolution of bacterial OMPs. Experiments are needed to confirm the observations, underlying hypothesis and application potentials.

## MATERIALS AND METHODS

### Bacterial species and strains

The previously annotated 39 representative *E. coli* or *Shigella* strains and 1 outgroup strain, *E. fergusonii* ATCC35469, were used in this study for screening of OMPs under mosaic evolution (23, 24). These representative strains have been successfully used for mosaic evolution analysis of 5 other OMPs, including FhuA, LamB, OmpA, OmpC, and OmpF (23, 24). To observe the distribution of HVR sequence types, all the 3,104 *E. coli* strains whose genomes were fully sequenced, assembled, and well annotated in the NCBI Genome database were selected, and their genome sequences and annotation files were downloaded (Data set S4). The genomes were all assembled with high quality and well annotated.

### Identification of *E. coli* OMP families

There are a lot of tools developed that could effectively predict bacterial OMPs, as reviewed in reference 13. However, many of the tools could not work currently. Finally, we selected two widely used tools, PRED-TMBB (37) and TMBETADISC-RBF (38), to predict OMPs from the genome-derived proteins of *E. coli* MG1655, using default settings. To further improve precision, the intersection of positive predictions from the two tools was considered the OMPs, with negative predictions but putative OMPs included, and positive predictions but putative non-OMPs excluded. Furthermore, BLASTp was implemented to search the homologs of the MG1655 OMPs in the other 39 *Escherichia* and *Shigella* strains. The parameters were set as ≥80% identity for an average length coverage of ≥90%.

### Screening of OMP families under mosaic evolution

For each OMP family, the nucleotide sequences encoding the proteins were analyzed for possible ingenic recombination with RDP4, a package widely applied for the detection of genetic recombinations (39). Ingenic recombination was defined if only at least one significant recombination event (*P* < 0.05) was identified by any one model or statistical approach integrated in RDP4. For each OMP family predicted by RDP4 with ingenic recombination, MEGA7.0 (40) was used to build the gene and protein trees with the neighbor-joining algorithm. The trees were manually checked and compared with the lineage tree based on the core proteome of the 40 *Escherichia* and *Shigella* strains, and thereby the OMP families were identified whose trees were apparently incongruent with the lineage tree. The core proteome and evolutionary analysis referred to previous reports (23, 24). Furthermore, for the OMP families whose gene and protein trees both deviated from the lineage tree, genetic exchange tests were conducted at both nucleic acid and protein levels. The positive observation of genetic exchange tests was defined to an OMP family for which at least one lineage showed a change from significance to non-significance in the Wilcoxon rank-sum tests for within-lineage vs inter-lineage genetic distance, or a reversal in direction at both nucleic acid and protein levels (24).

### Validation of mosaic evolution and HVR identification

A Python package, *HVRanalysis*, was developed to validate mosaic evolution and to identify the composition and sequence types of HVRs directly. We proposed three indices, MutIndex1, MutIndex2, and MutIndex, to comprehensively measure the local variation characteristics of proteins. In the first place, the protein sequences of an interesting family are aligned using CLUSTALW (41) with the default settings. Given *n* proteins, each with an aligned length *L*, peptide segments are continuously extracted by sliding a predefined window of width *l* and step size *s* across the aligned sequence of each protein simultaneously. For each set of *n* peptide segments obtained after each step, the number of sequence types *m* and the specific sequence types denoted as *S_1_*, *S*_2_, ..., *S_m_*, are analyzed after redundancy removal. The frequency *F_Si_* of each sequence type *S_i_* (*i* = *1*, *2*, ..., *m*) among the *n* peptide segments is calculated. In each window, the *n* peptide segments are observed site by site. A variation site is recorded whenever a different amino acid (or deletion) is found at the corresponding site in any sequence. The variant sites *v* are counted among the window length *l*.


(1) $$\text{ MutIndex1 }=\left(F_{S1}{}^{*}F_{S2}{}^{*}\ldots {}^{*}F_{Sm}\right)/(1/m{)}^{m}$$

(2)  MutIndex2 =v/l

(3) $$\text{ MutIndex }=\text{ MutIndex1 }{}^{*}\text{ MutIndex2 }$$

For each set of segments, MutIndex1, MutIndex2, and MutIndex are calculated according to the above formulas. MutIndex1 reflects the distribution balance of different sequence types within local regions within the strain population, MutIndex2 reflects the frequency of variations within local regions, and MutIndex combines MutIndex1 and MutIndex2 to comprehensively reflect the distribution of sequence types and variation sites. For each index, the mean and variance are calculated across the windows along the full protein alignment length, and a Gaussian distribution is simulated. A Kolmogorov-Smirnov (KS) test is performed to assess whether the distribution of the actual index conforms to or deviates from the simulated Gaussian distribution. If the KS test *P*-value is below the predefined significance level of 0.05, the corresponding index is not considered to follow a normal distribution along the protein alignment length, indicating the presence of local mosaicism. Continuous sites outside the 95% confidence interval of the simulated normal distribution are extracted, and the segments they compose are recorded as HVRs. The amino acid sequences of each HVR are extracted from the strains and deduplicated to obtain HVR sequence type or subtypes. An HVR sequence type was defined as the one with a sequence of more than two point substitutions or indels with the sequence of any other subtype. An HVR sequence subtype was defined as the one with the sequence of 1–2 point substitutions or indels with at least one other subtype.

*HVRanalysis* is freely accessible at http://61.160.194.165:3080/HVRanalysis. In this study, the default settings are used for *HVRanalysis* with *l* = 10 and *s* = 1 if not specified.

### Comparative genomic analysis

Each OMP under mosaic evolution is aligned against the proteins encoded by the genomes of various strains using BLASTp with identity >= 70% over an average length coverage of >= 70% to analyze the copy number of homologous genes in corresponding strains. The gene loci are delineated within the genome, and continuous nucleotide sequences including the gene sequences and the 25K flanking sequences at both upstream and downstream are retrieved. PipMaker (42) is used to perform collinearity analysis among the genes and their flanking sequences from different strains.

### Distribution analysis of HVR sequence types among *E. coli* population

The OMPs under mosaic evolution identified in 40 *Escherichia* and *Shigella* strains (termed as reference strains and OMPs) are used to determine and label their respective HVRs and sequence types based on the results of *HVR analysis*. The full-length protein sequences are aligned with BLASTp against the full proteome of 3,104 *E. coli* strains downloaded from NCBI Genome. For alignments with 100% identity across the full length, the corresponding *E. coli* strains, their best-matched reference OMPs, and the HVR sequence types and subtypes are annotated. For the orthologous OMPs of the remaining *E. coli* strains, an in-house Python script is prepared to search each known HVR sequence subtype so as to annotate the HVRs, HVR sequence types, and subtypes for the OMP orthologs in more strains. BLASTp is applied again to make mutual alignment for the unannotated OMP orthologs, followed by clustering the 100% identical ones. One representative protein is randomly selected from a cluster, multiple sequence alignments are performed to these representative OMPs and the reference OMPs, and the HVRs are annotated with new sequence types and subtypes identified according to the extent and patterns of variations.

### Micro-evolution analysis of the HVRs

Based on sequence homology, each HVR was classified into major sequence types (with >= 3 site mutations between each other). For a specific major HVR sequence type, the nucleic acid sequences were extracted from the corresponding OMPs of the reference *E. coli* strains, and selection analysis was performed using PAML (43). The Model 8 was used, omega was estimated for each codon site, and Naive Empirical Bayes (NEB) tests were performed to assess positive selection, that is, omega >1. The significance was preset as *P* > 0.95.

### Transmembrane topology and structural analysis of OMPs and HVRs

The signal peptides were predicted with SignalP 6.0 (44). DeepTMHMM (45) was used to predict the transmembrane topology of OMPs, while Phyre2 (46) and RoseTTAFold (47) were used to predict their tertiary structures. The DeepTMHMM prediction results were further manually confirmed or corrected by comparison with the experimentally resolved or predicted tertiary structures, as well as the literature annotations. The topological and structural characteristics of the regions where HVRs are located were annotated.

### Immunogenicity analysis of HVRs

ANTIGENpro (48) was used to analyze the antigenicity of the proteins representing each HVR sequence type. DiscoTope-2.0 (49) and CBTOPE (50) were used to analyze the structural B-cell epitopes. ABCpred (51) was used to analyze the linear B-cell epitopes. Besides, we also designed three new models, B_MM, BPBB, and JBFB, to predict linear B-cell epitopes based on different features and algorithms. JBFB showed the best performance and was, therefore, used in the study for antigen epitope analysis on the OMPs of different HVR sequence types. The default window size of 20 and predictive cutoff of 0 were used for JBFB prediction, by which a positive B-cell epitope of a length of 20 amino acids should have a prediction value of >0. The webserver, standalone models, and the documents are accessible freely through the link http://61.160.194.165:3080/B_pred.

## SUPPLEMENTAL MATERIAL

Data Set S1 aem.00557-25-s0001.xlsx Screening of *E. coli* OMPs under mosaic evolution.

Data Set S2 aem.00557-25-s0002.xlsx Annotation of the *E. coli* OMPs under mosaic evolution.

Data Set S3 aem.00557-25-s0003.xlsx Distribution of HVRs, sequence types, and sequence subtypes of the OMPs under mosaic evolution in extended size of *E. coli* strains.

Data Set S4 aem.00557-25-s0004.xlsx Information of *E. coli* strains with full genome sequences downloaded from the NCBI Genome database.

ASM does not own the copyrights to Supplemental Material that may be linked to, or accessed through, an article. The authors have granted ASM a non-exclusive, world-wide license to publish the Supplemental Material files. Please contact the corresponding author directly for reuse.
